# *Epilepsia Open*—March 2023—Announcements

**DOI:** 10.1002/epi4.12706

**Published:** 2023-03-01

## ILAE CONGRESSES

### 5th ILAE School on EEG in the First Year of Life: From newborn to Toddler

3–5 March 2023

Cochin, Kerala, India

Website: https://www.ilae.org/congresses/eeg‐in‐the‐first‐year‐of‐life2

### 12th ILAE School for Neuropathology and Neuroimaging in Epilepsy (INES 2023)

19–22 July 2023

Campinas, Brazil

Website: https://www.ilae.org/congresses/12th‐ilae‐school‐for‐neuropathology‐and‐neuroimaging‐in‐epilepsy‐ines‐2023

### 13th ILAE School for Neuropathology and Epilepsy Surgery

24–26 August 2023

Vienna, Austria

Website: https://www.ilae.org/congresses/13th‐ilae‐school‐for‐neuropathology‐and‐epilepsy‐surgery

### XVII Workshop on Neurobiology of Epilepsy (WONOEP 2023)

28 August–1 September 2023

Ireland

www.ilae.org/wonoep2023

### 35th International Epilepsy Congress

6 September 2023

Dublin, Ireland

www.ilae.org/iec2023

### 4th International Training Course on Neuropsychology in Epilepsy

8 March 2024

Lyon, France

Website: https://www.ilae.org/congresses/4th‐international‐training‐course‐on‐neuropsychology‐in‐epilepsy

### 15th European Epilepsy Congress

7–11 September 2024

Rome, Italy

Website: https://www.ilae.org/congresses/15th‐european‐epilepsy‐congress

## OTHER CONGRESSES

### International Conference on Artificial Intelligence in Epilepsy and Neurological Disorders

7–10 March 2023

Breckenridge, CO, USA

Website: https://www.aiepilepsy‐neuro.com/

### 12. Dreiländertagung 2023 – Joint Annual Meeting of the German and Austrian Societies of Epileptology and the Swiss Epilepsy League

15–18 March 2023

Berlin, Germany

Website: https://epilepsie‐tagung.de/

### International Congress on Structural Epilepsy & Symptomatic Seizures

29–31 March 2023

Gothenburg, Sweden

Website: http://www.stess2023.se/

### 11th Migration Course on Epileptology

15–19 June 2023

Belgrade, Serbia

Website: https://www.ilae.org/congresses/11th‐migration‐course‐on‐epileptology

### 15th European Pediatric Neurology Society Congress (EPNS): From genome and connectome to cure

20–24 June 2023

Prague, Czech Republic

Website: https://www.epns.info/epns‐congress‐2023/

### 9th Congress of the European Academy of Neurology

1–4 July 2023

Budapest, Hungary & Online

Website: https://www.ilae.org/congresses/9th‐congress‐of‐the‐european‐academy‐of‐neurology

### 19th Advanced San Servolo Epilepsy Course

17–28 July 2023

San Servolo (Venice), Italy

Website: https://www.ilae.org/congresses/19th‐advanced‐san‐servolo‐epilepsy‐course

### 20th International Congress of Neuropathology

13–16 September 2023

Berlin, German

Website: https://www.icn2023.de/

### ILAE British Branch Annual Scientific Meeting Gateshead

2–4 October 2023

Gateshead, United Kingdom

Website: https://www.ilae.org/congresses/ilae‐british‐branch‐annual‐scientific‐meeting‐gateshead

### 10th Eilat Educational Course: Pharmacological Treatment of Epilepsy

8–13 October 2023

Jerusalem, Israel

Website: https://www.eilatedu.com/

## 2024

### Seventeenth Eilat Conference on New Antiepileptic Drugs and Devices (EILAT XVII)

5–8 May 2024

Madrid, Spain

Website: https://www.ilae.org/congresses/seventeenth‐eilat‐conference‐on‐new‐antiepileptic‐drugs‐and‐devices‐eilat‐xvii

### 10th Congress of the European Academy of Neurology

29 June–2 July 2024

Helsinki, Finland

Website: https://www.ilae.org/congresses/10th‐congress‐of‐the‐european‐academy‐of‐neurology

